# Supplementary material for: Low Serum Vitamin D Status Is Associated with Incident Alzheimer’s Dementia in the Oldest Old
Source: Nutrients. 2022 Dec 23;15(1):61. doi: 10.3390/nu15010061 (PMC9824107; doi:10.3390/nu15010061)
Supplement: Supplementary file 1 [file nutrients-15-00061-s001.zip › nutrients-2085506-supplementary.pdf]

Table S1: Characteristics of the study participants

	Main sample<br>n= 1,334
Age (years)	84 ± 3
Female (n, %)	861 (64.5)
BMI (kg/m^2)	25.9 ± 3.7
Missing values (n, %)	30 (2.2)
APOE ε4 allele (n, %)	248 (18.6)
Missing values (n, %)	44 (3.3)
Time to develop Alzheimer's dementia (years)	3 ± 2
Time to censoring (years)	5 ± 2
Blood analyses	
Vitamin A (mg/L)	0.54 ± 0.23
Beta-carotene (mg/L)	0.32 (IQR: 0.22-0.48)
Vitamin D (nmol/L)	37.0 (IQR: 24.8-58.3)
Vitamin E (mg/L)	15.73 ± 6.33
Creatinine (mg/dl)	1.00 (IQR: 0.83-1.22)
Missing values (n, %)	30 (2.2)
Total cholesterol (g/L)	2.21 ± 0.47
Missing values (n, %)	30 (2.2)
Triglycerides (g/L)	1.12 (IQR: 0.87-1.50)
Missing values (n, %)	269 (20.1)
Education (n, %)	
Lower	779 (58.4)
Middle	396 (29.7)
High	159 (11.9)

(Continued) **Table S1:** Characteristics of the study participants

Physical activity (n, %)	
Low (0-1)	111 (8.3)
Middle (2)	892 (66.9)
High (3-5)	303 (22.7)
Missing values (n, %)	28 (2.1)
Smoking (n, %)	
Never	681 (51.0)
Past	569 (42.7)
Current	84 (6.3)
Vitamin supplement intake (n, %)	79 (5.9)
Missing values (n, %)	21 (1.6)
Systolic blood pressure (mmHg)	136 ± 16
Missing values (n, %)	74 (5.5)
Diastolic blood pressure (mmHg)	80 (IQR: 70-80)
Missing values (n, %)	74 (5.5)
ACE inhibitors usage (n, %)	47 (3.5)
Calcium channel blockers usage (n, %)	5 (0.4)
Ginkgo biloba usage (n, %)	10 (0.8)
Laxative usage (n, %)	9 (0.9)

Values are means ± SD, numbers (valid percentages), or medians (interquartile range). Abbreviations: AgeCoDe, German Study on Ageing, Cognition and Dementia in Primary Care Patients; APOE ε4, apolipoprotein ε4; g, grams; n, number; mmHg, millimeter of mercury.

Table S2. Specification of the multiple imputation procedure

Software	IBM SPSS Statistics for Windows (Release 21.0.0.1)
Imputation method	Fully conditional specification (Markov chain Monte Carlo method)
Key settings	Maximum iterations: 20
Imputed data sets	10
Variables included in the imputation procedure (imputed or used as predictors of missing	Dementia at baseline; age at baseline; participated in follow up 3 vitamin A (mg/L); Beta-carotene (mg/L); vitamin D (mmol/L); vitamin E (mg/L); CERAD (baseline-FU3); Follow up 3 aggregated: Healthy controls, MCI, dementia variable; Incident Alzheimer's dementia FU8; incident dementia FU8; vascular dementia FU8; time variable; sex; age at FU3; Apolipoprotein e4; education; body weight (kg); height (cm); smoking; creatinine; cholesterol; triglycerides; systolic blood pressure; diastolic blood pressure; depression; physical activity; vitamin supplement intake; month of blood drawing.
Additionally added predictive variables to increase plausibility of missing at random	body weight at FU4 (kg); height at FU4 (cm); ability to conduct chorus around the house FU3; hypertension FU3; diabetes mellitus type I and II FU3; kidney failure FU3; lipids and cholesterol combined FU3; hypertension FU3; medication use FU2 and FU3
Not normally distributed variables were treated with:	Predictive mean matching
Binary/categorical variables were treated with:	Logistic regression models

Abbreviations: FU= follow up visit

Table S3: Longitudinal associations between blood (serum/plasma) concentrations of vitamins A, D and E and beta-carotene and incident vascular dementia over a 7-year follow up period (n=1,334)

	Incident vascular dementia (n=41 cases)			
	Model 1		Model 2	
**Vitamins**	*HR* (95 % CI)	*P*	*HR* (95 % CI)	*P*
Vitamin A (mg/L)	1.84 (0.61; 5.54)	0.276	2.19 (0.77; 6.30)	0.144
Beta-carotene (mg/L)	1.01 (0.37; 2.75)	0.992	1.21 (0.44; 3.30)	0.711
Vitamin E (mg/L)	0.98 (0.93; 1.03)	0.376	0.98 (0.92; 1.04)	0.469
Vitamin D (mmol/L)	1.01 (0.99; 1.02)	0.324	1.01 (0.99; 1.02)	0.461
**Vitamin D cut-offs**				
≥50 (mmol/L) (n=449)	*Reference*		*Reference*	
≥25 -<50 (mmol/L) (n= 548)	0.30 (0.13; 0.71)	0.006	0.31 (0.12; 0.78)	0.013
<25 (mmol/L) (n= 337)	0.96 (0.48; 1.91)	0.910	0.91 (0.38; 2.15)	0.823

Based on imputed data. Abbreviations: *APOE* ε4, apolipoprotein ε4; CI= confidence interval; HR, hazard ratio. Model 1 is adjusted for age, sex, *APOE* ε4 and education. Model 2 is adjusted as for model 1, plus BMI, physical activity and smoking, memory decline before study baseline, total cholesterol, creatinine, systolic blood pressure, diastolic blood pressure and vitamin supplement intake (in addition for vitamin D: vitamin D supplement intake and month of blood draw). A P-value <0.05 is considered to be statistically significant.

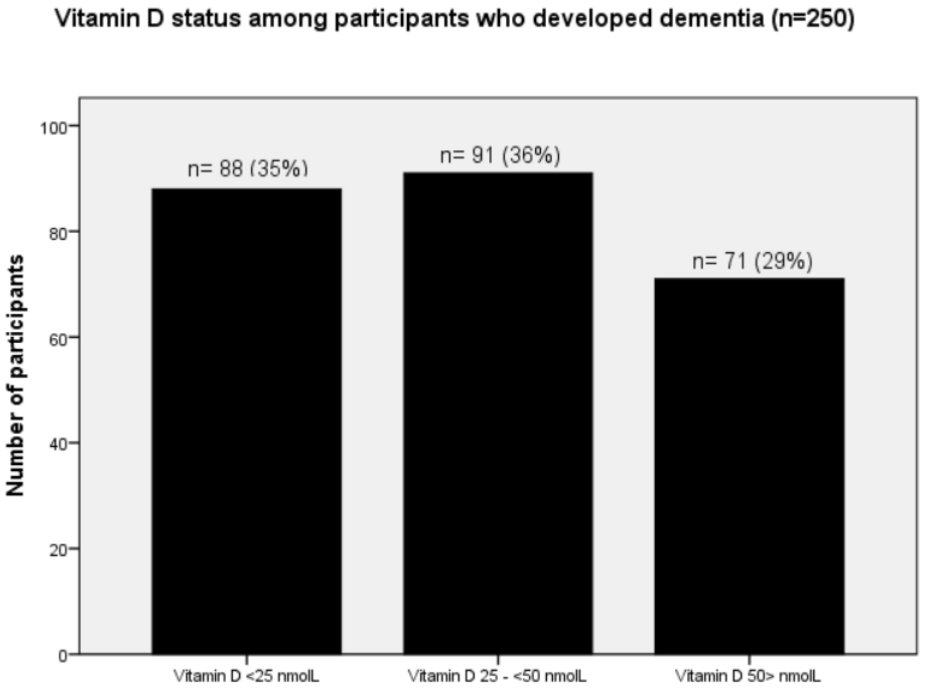

Figure S1. Vitamin D status at visit 3 among participants who developed all-cause dementia during follow-up.

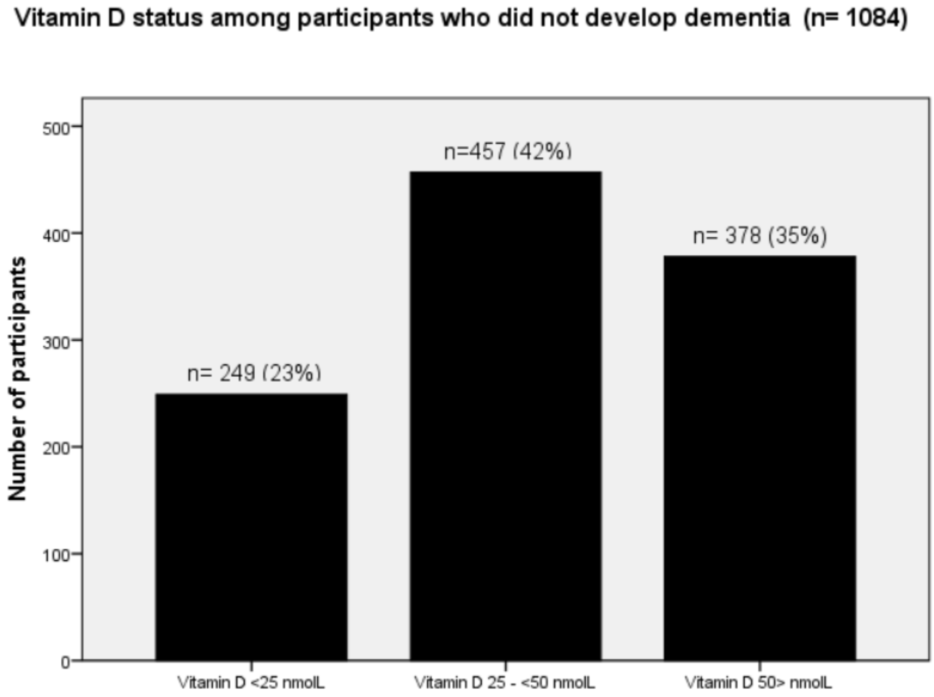

Figure S2. Vitamin D status at visit 3 among participants who did not develop all-cause dementia during follow-up.
